# Ensemble outlier detection and gene selection in triple-negative breast cancer data

**DOI:** 10.1186/s12859-018-2149-7

**Published:** 2018-05-04

**Authors:** Marta B. Lopes, André Veríssimo, Eunice Carrasquinha, Sandra Casimiro, Niko Beerenwinkel, Susana Vinga

**Affiliations:** 1 IDMEC, Instituto Superior Técnico, Universidade de Lisboa, Av. Rovisco Pais 1, Lisboa, 1049-001 Portugal; 2 Luis Costa Lab, Instituto de Medicina Molecular, Faculdade de Medicina da Universidade de Lisboa, Avenida Professor Egas Moniz, Lisboa, 1649-028 Portugal; 3 Department of Biosystems Science and Engineering, ETH Zurich, Mattenstrasse 26, Basel, 4058 Switzerland; 4 SIB Swiss Institute of Bioinformatics, Mattenstrasse 26, Basel, 4058 Switzerland; 5 INESC-ID, Instituto de Engenharia de Sistemas e Computadores - Investigação e Desenvolvimento, Rua Alves Redol 9, Lisboa, 1000-029 Portugal

**Keywords:** Ensemble modeling, High-dimensionality, Outlier detection, Rank Product test, Triple-negative breast cancer

## Abstract

**Background:**

Learning accurate models from ‘omics data is bringing many challenges due to their inherent high-dimensionality, e.g. the number of gene expression variables, and comparatively lower sample sizes, which leads to ill-posed inverse problems. Furthermore, the presence of outliers, either experimental errors or interesting abnormal clinical cases, may severely hamper a correct classification of patients and the identification of reliable biomarkers for a particular disease. We propose to address this problem through an ensemble classification setting based on distinct feature selection and modeling strategies, including logistic regression with elastic net regularization, Sparse Partial Least Squares - Discriminant Analysis (SPLS-DA) and Sparse Generalized PLS (SGPLS), coupled with an evaluation of the individuals’ outlierness based on the Cook’s distance. The consensus is achieved with the Rank Product statistics corrected for multiple testing, which gives a final list of sorted observations by their outlierness level.

**Results:**

We applied this strategy for the classification of Triple-Negative Breast Cancer (TNBC) RNA-Seq and clinical data from the Cancer Genome Atlas (TCGA). The detected 24 outliers were identified as putative mislabeled samples, corresponding to individuals with discrepant clinical labels for the HER2 receptor, but also individuals with abnormal expression values of ER, PR and HER2, contradictory with the corresponding clinical labels, which may invalidate the initial TNBC label. Moreover, the model consensus approach leads to the selection of a set of genes that may be linked to the disease. These results are robust to a resampling approach, either by selecting a subset of patients or a subset of genes, with a significant overlap of the outlier patients identified.

**Conclusions:**

The proposed ensemble outlier detection approach constitutes a robust procedure to identify abnormal cases and consensus covariates, which may improve biomarker selection for precision medicine applications. The method can also be easily extended to other regression models and datasets.

**Electronic supplementary material:**

The online version of this article (10.1186/s12859-018-2149-7) contains supplementary material, which is available to authorized users.

## Background

The rising of genome sequencing technology has advanced biomedical science into precision medicine, under the premise that molecular information improves the accuracy with which patients are categorized and treated [1]. This is particularly important for cancer, with similar histopathology showing differential clinical outcome, and treatments failing essentially because of varying tumor genotype and phenotypic behavior in each individual [2].

Cancer genomics refers to the study of tumor genomes using various profiling strategies including (but not limited to) DNA copy number, DNA methylation, and transcriptome and whole-genome sequencing - technologies that may collectively be defined as omics [3]. The resulting omics’ data allows not only a more in-depth knowledge on the cancer biology, but also the identification of diagnostic, prognostic, and therapeutic markers that will ultimately improve patient outcomes [3]. Cancer genomics therefore holds the promise of playing an important role towards precision cancer management.

However, this flood of ’omics data also brings many challenges when learning regression models: first, genomic datasets are high-dimensional, corresponding to measurements of thousands genes (the *p* covariates) for each individual, often highly correlated and outnumbering the cases enrolled for the study, *N*. In fact, this crucial *N*≪*p* or high-dimensional problem, which occurs very frequently in patientomics data, may cause instability in the selected driver genes and poor performance of predictive models [4]; second, genomic data usually contain abnormal variable measurements arriving from many sources (e.g., experiment errors), which might be regarded as potential outliers that may end-up in a incorrect labeling/classification of the patients and, consequently, precipitate failure in the cancer treatment. On the other hand, abnormal observations that are not wrongly classified, might represent interesting clinical cases that can potentially disclose crucial information on the biology of cancer. In both cases, outlier patients must be identified, so that further investigation on these patients is undertaken. Variable selection and outlier detection are therefore key steps when fitting regression models to cancer genomic data.

We will address these problems through an ensemble or consensus outlier detection approach, focusing on the classification of high-dimensional patientomics data. Ensemble analysis has been widely explored for classification (e.g. by *boosting*, *bagging* or *random forests*), but rather limited in the outlier detection context [5]. For instance, Lazarevic and Kumar [6] developed a *feature bagging* outlier ensemble to detect outliers in high-dimensional and noisy datasets, based on randomly selected feature subsets from original feature sets. Motivated by *random forests*, Liu et al. [7] proposed *isolation forest* for anomaly detection.

Since multiple classification and dimensionality reduction strategies exist, ranging from variable selection by regularized optimization, to feature extraction e.g. by Partial Least Squares (PLS) regression, and also many outlier detection methods have been proposed, based on distinct residual measures, our approach will be to gather these different results into a unique ranking for the most outlier observations. This is achieved with the application of the rank product (RP) test, a well-known statistical technique previously used in detecting differentially regulated genes in replicated microarray experiments [8] and outlying patients in survival data [9]. It has also shown to support meta-analysis of independent studies ([10]).

The proposed model-based outlier detection procedure provides a structured framework to separate abnormal cases, i.e., those significantly deviating from what is expected given a specific model. The definition of outlier becomes, therefore, highly coupled with the model statistical learning process, with the obvious interpretability advantage: an outlier is a case that deviates from what would be expected given the corresponding covariates, across several modeling selection strategies. As deviances are dependent on the model chosen, with an observation deviating from a given model not deviating from another, our ensemble approach is expected to correct for the specific uncertainty each model brings. The rationale is that if a given observation is systematically classified as an outlier independently of the chosen model, there is evidence for being a true discrepant observation given its covariates.

To illustrate the application of the proposed procedure, the Breast Invasive Carcinoma (BRCA) dataset publicly available from the Cancer Genome Atlas (TCGA) Data Portal (https://cancergenome.nih.gov/) was used. From the BRCA dataset, we focused on a specific type of breast cancer, the Triple-Negative Breast Cancer (TNBC), which is the most heterogeneous group of breast cancers, presenting a significantly shorter survival following the first metastatic event when compared with those with non-basal-like/non-triple-negative controls [11]. It is characterized by lack of expression of estrogen receptor (ER), progesterone receptor (PR), and human epidermal growth factor receptor type 2 (HER2) [12]. Endocrine and HER2-targeted therapies are therefore not successful, which fosters the identification of new biomarkers and potential druggable targets for effective clinical management of TNBC.

Classifying patients into ‘positive’ or ‘negative’ for the presence of these receptors is a key step in therapy decision. It has been reported that up to 20% of immunohistochemical (IHC) ER and PR determinations worldwide might be inaccurate (false negative or false positive), mainly due to variation in preanalytic variables, thresholds for positivity, and interpretation criteria [13]. The obvious consequences are false negatives being not eligible for endocrine therapy, thus not benefiting from it, and failure of hormonal therapy in false positives. Regarding HER2, while a false positive HER2 assessment, either by IHC or fluorescence in-situ hybridization (FISH) testing, leads to the administration of potentially toxic, costly and ineffective HER2-targeted therapy, a false negative HER2 assessment results in denial of anti-HER2 targeted therapy for a patient who could benefit from it [14]. Accurate test performance following the published guidelines [13, 14] is thus crucial, as it will determine the success of the applied therapy. To overcome uncertainty in variables assessment, appropriate outlier detection methods stand as invaluable tools in personalized cancer management. Whenever an observation is detected as influential, a careful inspection on its gene expression profile should be conducted and, if appropriate, further re-testing for the critical variables under studied is warrant.

In this work we measure the outlierness (the degree of deviation) of breast cancer patients (TNBC and non-TNBC) in either selected subsets of covariates or projections of data into subspaces of reduced dimension. The goal is to identify the observations that are systematically classified as influential (thus potential outliers), independently of the model chosen. Three strategies for data dimension reduction were considered and will be described below: i) variable selection by sparse logistic regression using elastic net (EN) regularization; ii) variable selection and feature extraction by Sparse PLS Discriminant Analysis (SPLS-DA); and iii) variable selection and feature extraction by Sparse Generalized PLS (SGPLS). For each method the ranks of influential observations detected were obtained and combined for a consensus outlier detection by the RP test.

In conclusion, the goals of the ensemble method proposed are two-fold: i) the detection of outlier observations that deviate from the classification model learnt can pinpoint to potential mislabeling of the original TCGA clinical data; and ii) the identification of a consensus set of genes that may play a role in TNBC management.

## Methods

### Classification and dimensionality reduction

When the goal is to build a predictive model based on high-throughput genomic data for assessing a clinical binary outcome of a patient, e.g., ‘cancer’ vs. ‘normal’ *status*, or different types of cancer, logistic regression is a common choice. Binary logistic regression is a popular classification method that describes the relationship between one or more independent variables and a binary outcome variable, which is given by the logistic function

1 $$ p_{i} = Prob \left(Y_{i}=1\right)=\frac{\exp\left(\mathbf{x}_{i}^{T} \boldsymbol{\beta}\right)}{1+\exp\left(\mathbf{x}_{i}^{T} \boldsymbol{\beta}\right)},  $$

where *X* is the *n*×*p* design matrix (*n* is the number of observations and *p* is the number of covariates or features), *p*_*i*_ is the probability of success (i.e., *Y*_*i*_=1) for observation *i* and ***β***=(*β*_1_,*β*_2_,…,*β*_*p*_) are the regression coefficients associated to the *p* independent variables. This is equivalent to fitting a linear model in which the dependent variable (clinical outcome) is replaced by the logarithm of the odds ratio (defined as the ratio of the probability of success, *p*_*i*_, and the probability of failure, 1−*p*_*i*_), through the *logit* transformation given by

2 $$ \log \left(\frac{p_{i}}{1-p_{i}}\right) = \mathbf{x}_{i}^{T} \boldsymbol{\beta}.  $$

It is therefore assumed that the *logit* transformation of the outcome variable has a linear relationship with the predictor variables. The parameters of the logistic model are estimated by maximizing the log likelihood function of the logistic model given by

3 $$  l\left(\boldsymbol{\beta}\right) = \sum_{i=1}^{n} \left\{y_{i} \mathbf{x}_{i}^{T}\boldsymbol{\beta} - \log \left(1+ e^{\mathbf{x}_{i}^{T}\boldsymbol{\beta}}\right)\right\}.  $$

Variable selection is extremely important in cancer genomics, owing to the identification of biomarkers associated to the disease or its subcategories. The inherent high-dimensionality and multi-collinearity of patientomics data, with variables very often outnumbering the cases enrolled, constitutes a challenge to identify an interpretable model since it usually leads to ill-posed inverse problems. In this context, regularized optimization is a promising strategy to cope with this problem, promoting the selection of a subset of variables while learning the model.

Several regularization methods have been proposed for variable selection in high-dimensional data, namely, the *least absolute shrinkage and selection operator* (LASSO) [15], using a *l*_1_ regularizer, Ridge regression, which shrinks the estimated coefficients towards zero by using a *l*_2_−*norm* penalty, and the elastic net (EN) [16], where the regularizer is a linear combination of *l*_1_ and *l*_2_ penalties.

The EN penalty is controlled by *α*, as follows

4 $$ \boldsymbol{\hat \beta} = \arg\min_{\boldsymbol{\beta}} \Vert Y - \mathbf{X} \boldsymbol{\beta} \Vert^{2} + \lambda \left\{(1-\alpha) \Vert \boldsymbol{\beta} \Vert^{2}_{2}/2 + \alpha \Vert \boldsymbol{\beta} \Vert_{1}\right\},  $$

with *α*=1 corresponding to LASSO and *α*=0 to ridge, and the tuning parameter *λ* controlling the strength of the penalty. While in the presence of highly correlated variables the LASSO tends to arbitrarily select one of those variables, EN encourages *β*_*i*_ to be close to *β*_*j*_ for highly correlated variables, therefore inducing variables group formation. Feature grouping is particularly important in the context of modeling gene expression data, as highly correlated genes shall be kept as a group and not arbitrarily discarded.

The problem of multicollinearity can be approached by feature extraction methods like partial least squares (PLS) regression [17, 18]. In PLS regression an orthogonal basis of *latent variables* (LV) – not directly observed or measured – is constructed in such a way that they are maximally correlated with the response variable. The basic assumptions of PLS regression is that the relationship between **X** and **Y** is linear and that this linearity assumption still holds for the relationship between the latent variables.

Formally, PLS expresses **X**(*n*×*p*) and **Y**(*n*×*m*) as **X**=**TP**^*T*^+***E*** and **Y**=**UQ**^*T*^+***F***, where **T** and **U** are the (*n*×*L*) matrices of the *L* extracted score (latent) vectors (*L*≪*p*), whereas **P** (*p*×*L*) and **Q** (*m*×*L*) are the matrices of orthogonal loadings, and **E** (*n*×*p*) and **F** (*n*×*m*) are matrices of residuals. The latent components **T** are defined as **T=XW**, where **W**(*p*×*L*) are *L* direction vectors (1≤*L*≤ min{*n*,*p*}). Given **T** and **U**, the PLS estimate of the regression coefficients vector ***β***=(*β*_1_,…,*β*_*p*_) is

5 $$ \hat{\boldsymbol{\beta}}= \mathbf{X}^{T}\mathbf{U}\left(\mathbf{T}^{T}\mathbf{X}\mathbf{X}^{T}\mathbf{U}\right)^{-1} \mathbf{T}^{T} \mathbf{Y}.  $$

The *l*-th direction vector $\hat {\mathbf {w}}_{l}$ is obtained by solving:

6 $$  \max_{\mathbf{w}} \ \left\{\mathbf{w}^{T} \mathbf{Mw}\right\},  $$

subject to **w**^*T*^**w**=1 and $\mathbf {w}^{T} \mathbf {S_{XX}} \hat {\mathbf {w}}_{l} = 0 (s = 1,\dots,l-1)$, where **M**=**X**^*T*^**YY**^*T*^**X** and *S*_*XX*_ represents the sample covariance matrix of the predictors.

The projection of the observed data onto a subspace of orthogonal LVs, typically of small number, has been shown to be a powerful technique when the observed variables are highly correlated, noisy, and the ratio between the number of observations and variables is small, which justifies its use for the analysis of genomic data [19].

Modern genomic data analysis involves a high number of irrelevant variables, yielding inconsistency of coefficient estimates in the linear regression. Chun and Keles [20] proposed sparse partial least squares (SPLS) regression, which imposes sparsity when constructing the direction vectors, thereby the resulting LVs only dependent on a subset of the original set of predictors. SPLS incorporates variable selection into PLS by solving the following minimization problem instead of the original PLS formulation in Eq. 6

7 $$ \begin{aligned} \min_{\mathbf{w},\mathbf{c}} \ \Big\{&-l\mathbf{w}^{T} \mathbf{Mw} + (1-l) \left(\mathbf{c} - \mathbf{w}\right)^{T}\\ &\times \mathbf{M}\left(\mathbf{c} - \mathbf{w}\right) + \lambda_{1} \Vert \mathbf{c} \Vert_{1} + \lambda_{2} \Vert \mathbf{c} \Vert_{2}\Big\}, \end{aligned}  $$

subject to **w**^*T*^**w**=1, where **M**=**X**^*T*^**YY**^*T*^**X**. This formulation promotes sparsity by imposing *l*_1_ penalty onto a surrogate of direction vector (**c**) instead of the original direction vector (**w**), while keeping **w** and **c** close to each other. The *l*_2_ penalty takes care of the potential singularity of **M** [21].

PLS can also be applied to classification problems, when the response variable is categorical and expresses a class membership. Chung and Keles [21] proposed two methods extending SPLS to classification. The first, SPLS Discriminant Analysis (SPLS-DA), is a two-stage procedure. In a first step, SPLS regression is used to construct LVs by treating the categorical response as a continuous variable (for a binary response a dummy {0,1} code is used). In the second step, given the number of LVs, *L*, is usually much smaller than the sample size *n*, a linear classifier such as linear discriminant analysis (LDA) and logistic regression is applied. The second method extends SPLS to the Generalized framework, herein called SGPLS. The minimization problem in Eq. 3 can be solved with the Newton-Raphson algorithm which results in the iteratively re-weighted least squares (IRLS) [21]. SPLS can be incorporated into the GLM framework by solving this weighted least squares problem

8 $$ \min_{\boldsymbol{\beta}} \sum_{i=1}^{n} v_{i} \left(z_{i} - \mathbf{x}_{i}^{T} \boldsymbol{\beta}\right)^{2},  $$

where *v*_*i*_=*p*_*i*_(1−*p*_*i*_) and $z_{i} = \mathbf {x}_{i}^{T} \boldsymbol {\beta } + \left (y_{i} - p_{i}\right)/v_{i}$ (the working response). The direction vectors of SGPLS are obtained by solving Eq. 7 subject to **w**^*T*^**w**=1, where **M**=**X**^*T*^**Vzz**^*T*^**VX**, with **V** the diagonal matrix with entries *v*_*i*_, and **z**=(*z*_1_,…,*z*_*n*_) the vector of working responses.

Dimensionality reduction is a critical step before outlier detection, as working on the full space hampers the disclosure of outliers hidden in subspace projections. Outlier inspection can be firstly approached via graphical examination of the residuals. Residuals are the differences between the predicted and actual values. There are several types of residuals, e.g., Pearson and deviance, along with their standardized versions. An outlier is an observation with a large residual, whose dependent variable value is unusual given its value on the predictor variables; an outlier may indicate a sample peculiarity or a data entry error. A leverage observation, on the other hand, is an observation with an extreme value on a predictor variable. Leverage is a measure of how far an independent variable deviates from its mean. High leverage observations can have a great amount of effect on the estimate of the regression coefficients. Influence can be thought of as the product of leverage and outlierness. In this context, the Cook’s distance, *D* [22, 23], is a measure of influence widely used in outlier detection that combines the information of leverage and residual. For each observation *i*, *D*_*i*_ measures the change in $\hat {Y}$ for all observations with and without observation *i*, so that we know how much the observation *i* impacted the fitted values:

9 $$ D_{i} = \frac{r_{i}^{2} \ h_{ii}}{1 - h_{ii}},  $$

with *r*_*i*_ denoting the standardized Pearson residual given by

10 $$ r_{i} = \frac{y_{i} -\hat{y_{i}}}{\sqrt{p_{i} \left(1 - p_{i}\right)} \sqrt{1 - h_{ii}}},  $$

and *h*_*ii*_ the *i*th diagonal element of the matrix **H**, defined for logistic regression as

11 $$ \mathbf{H} = \mathbf{V}^{1/2}\mathbf{X}\left(\mathbf{X}^{T}\mathbf{V}\mathbf{X}\right)^{-1}\mathbf{X}^{T}\mathbf{V}^{1/2}  $$

where **V** is a *n*×*n* diagonal matrix with general element *p*_*i*_(1−*p*_*i*_) [24, 25], as described above.

Variables disclose outlying observations independently. Depending on the data dimension reduction strategy used, different outlier sets might emerge, as an individual deviating in a particular subspace of variables may look fairly normal in the other subspaces evaluated. Given a number of outlierness rankings based on an influential measure (e.g., the Cook’s distance) obtained from different modelling strategies, a consensus ranking of observations is thus desired.

The performance of model-based outlier detection tools can be significantly improved if combined into an outlier ensemble. The rationale behind ensemble learning is to combine different predictions by multiple learning processes into a more accurate prediction, which becomes particularly useful in the presence of multiple models yielding different sets of outliers. The RP test has been used in the context of outlier ensemble analysis, providing a consensus ranking of all observations ranked by their level of outlierness, given a set of models or influence measures.

### The rank product (RP) test

The Rank Product (RP) is a non-parametric statistical technique that allows the statistical assessment of consensus rankings obtained in distinct experiments. Given different modeling strategies lead to different sets of influential observations based on a given measure of outlierness, the application of RP tests in the present work aims at identifying the observations that are consistently classified as influential, irrespectively of the specific model chosen. This procedure thus constitutes a consensus approach to outlier detection.

Given *D*_*ij*_ the Cook’s distance (the measure of outlierness used in this work) of the *i*^*th*^ observation (*i*=1,…,*n*) obtained by the *j*^*th*^ model, the deviance rank for *D*_*ij*_ considering model *j* (*j*=1,…,*k*) is defined by *R*_*ij*_=*rank*(*D*_*ij*_), with 1≤*R*_*ij*_≤*n*. The lower the rank, the larger the deviance, i.e., the more outlying the observation is. The RP is defined as $RP_{i}=\prod _{j=1}^{k}R_{ij}$. After ranking the observations by their RP, their corresponding *p*-values, under the null hypothesis that each individual ranking is uniformly distributed, are obtained. The statistical significance of *RP*_*i*_ under the null hypothesis of random rankings was obtained following Heskes et al. [26], based on the geometric mean of upper and lower bounds, defined recursively.

When many observations are tested, type-I errors (false positives) increase. The False Discovery Rate (FDR) [27], which is the expected proportion of false positives among all tests that are significant, is an example of a correction method dealing with the multiple testing problem. FDR sorts in an ascendant order the *p*-values and divides them by their percentile rank. The measure used to determine the FDR is the *q*-value. For the *p*-value, an *α* level of 0.05 implies that 5% of all tests will result in false positives under the null hypothesis, instead, for the *q*-value, 0.05 implies that 5% of significant tests will result in false positives. The *q*-value is therefore able to control the number of false discoveries in those tests.

### Triple-negative breast cancer (TNBC) data

The BRCA RNA-Seq Fragments Per Kilobase per Million (FPKM) dataset was imported using the ‘brca.data’ R package (https://github.com/averissimo/brca.data/releases/download/1.0/brca.data_1.0.tar.gz). The BRCA gene expression data is composed of 57251 variables for a total of 1222 samples from 1097 individuals. From those individuals, 1102 presented with a primary solid tumor, 7 with metastases, and for 113 normal breast tissue was obtained. Only samples from primary solid tumor were considered for analysis. A subset of 19,688 variables, including the three TNBC-associated key variables ER (ENSG00000091831), PR (ENSG00000082175) and HER2 (ENSG00000141736), was considered for further analysis, corresponding to the protein coding genes reported from the Ensembl genome browser [28] and the Consensus CDS [29] project.

The TNBC data was built from the BRCA dataset. The TNBC binary response vector **Y** was created, with ‘1’ corresponding to TNBC individuals (with ER, PR and HER2 ‘negative’), and non-TNBC (‘0’) to non-TNBC patients, whenever at least one of the three genes is *positive*.

The individuals’ *status* regarding ER, PR and HER2, needed for building **Y**, were obtained from the clinical data, composed of 114 variables. However, for HER2, three possible variable sources were available, corresponding to the HER2 (IHC) level, HER2 (IHC) *status* and HER2 (FISH), often providing distinct HER2 labels. For instance, an inspection on the classification of individuals into HER2 (IHC) levels and HER2 (IHC) *status* (Table 1) revealed non-concordance for HER2 classification (‘positive’ vs. ‘negative’) for 13 individuals (highlighted in bold). Also, 15 individuals showed non-concordance between HER2 (IHC) *status* and HER2 (FISH).

**Table 1:** Correspondence (number of cases) between the HER2 classification of individuals by IHC level and *status*, and FISH, obtained from the BRCA clinical data (individuals with non-concordance for HER2 classification by different testing (‘positive’ vs. ‘negative’) are highlighted in bold)

		HER2 (IHC)*status*					
		‘’	Equivocal	Indeterminate	Negative	Positive	Total
HER2 (IHC) level	‘’	176	7	6	241	41	471
	0 (negative)	0	0	0	60	0	60
	1+ (negative)	0	4	1	255	**11**	271
	2+ (indeterminate)	0	166	4	1	27	198
	3+ (positive)	1	1	1	**2**	85	90
	Total	177	178	12	559	164	1090
HER2 (FISH)	‘’	115	18	4	432	104	673
	equivocal	0	3	0	0	2	5
	indeterminate	0	0	0	3	1	4
	negative	53	138	6	121	**12**	330
	positive	9	19	2	**3**	45	78
	Total	177	178	12	559	164	1090

Table 2 shows the gene expression of three TNBC-associated variables for individuals with discordant HER2 (IHC) *status* and HER2 (IHC) level classifications. This is particularly important for individuals with both ER and PR ‘negative’ based on the clinical variables (highlighted in bold), as the HER2 labeling will determine the final classification of patients into TNBC vs. non-TNBC, which will be distinct (potential outlier), depending on the HER2 label chosen.

**Table 2:** Individuals with discordant HER2 (IHC) *status* and level, not measured by FISH (individuals not expressing ER and PR, and without a FISH classification are highlighted in bold)

Individual	ER	PR	HER2	HER2 level (IHC)	HER2 *status* (IHC)	Type
TCGA-AC-A8OS	94.72(+)	1.14(+)	23.55	1+	+	non-TNBC
**TCGA-LL-A73Y**	**0.22(-)**	**0.18(-)**	**19.00**	**3+**	**-**	**TNBC**
^∗^ **TCGA-AN-A0FL**	**0.09 (-)**	**1.7(-)**	**15.07**	**1+**	**+**	**non-TNBC**
^∗^ **TCGA-AN-A0FX**	**1.13(-)**	**0.64(-)**	**24.02**	**1+**	**+**	**non-TNBC**
TCGA-AN-A0FK	128.26(+)	31.59(+)	25.62	1+	+	non-TNBC
TCGA-E9-A295	17.36(+)	6.80(+)	34.83	1+	+	non-TNBC
^∗^TCGA-AC-A3YI	5.65(+)	0.76(+)	60.87	1+	+	non-TNBC
TCGA-JL-A3YW	0.35(+)	0.09(+)	31.47	1+	+	non-TNBC
TCGA-AN-A0FS	91.87(+)	1.16(-)	43.92	1+	+	non-TNBC
TCGA-AN-A0FN	21.34(+)	1.14(+)	17.50	1+	+	non-TNBC
^∗^TCGA-AN-A0FJ	0.08(+)	0.04(-)	14.28	1+	+	non-TNBC
TCGA-AC-A3W6	28.89(+)	0.26(+)	19.24	3+	-	non-TNBC
TCGA-AN-A03X	0.75(+)	12.28(+)	38.03	1+	+	non-TNBC

Individuals marked with asterisks show no concordance regarding HER2 labeling by different testing and are misclassified by logistic regression based on the 3 variables clinically used to classify breast cancer patients into TNBC

Individuals with discrepant labels regarding the HER2 (IHC) *status* and HER2 (FISH) can be found in Table 3. For those not expressing ER and PR, based on the clinical variables, and with different HER2 *status* and FISH (highlighted in bold), a distinct response value (‘1’ or ‘0’, *i.e*, TNBC and non-TNBC, respectively) can be produced, depending on the HER2 method chosen. Therefore, when building the response vector **Y**, care must be taken as discrepant individual classifications between the different methods for HER2 determination occur, and the variable chosen will determine the final TNBC individual classification. Individuals with non-concordant HER2 testing results might be regarded as possibly mislabeled samples, herein called *suspect individuals*, which are potential outliers. Special attention to these individuals with discrepant classification will be taken during discussion, by assessing if they are influential observations detected by the procedure and by analysing their covariates in detail.

**Table 3:** Individuals with discordant HER2 (IHC) *status* and HER2 (FISH) classification (individuals not expressing ER and PR are highlighted in bold)

Individual	ER	PR	HER2	HER2 *level*	HER2 *status*	HER2	HER2 *status*	Type
				(IHC)	(IHC)	(FISH)	(IHC + FISH)	
^∗^TCGA-LL-A5YP	0.16(+)	0.05(-)	15.10	1+	-	+	+	non-TNBC
^∗^ **TCGA-A2-A0EQ**	**2.13(-)**	**0.04(-)**	**30.5**	**3+**	**+**	**-**	**-**	**TNBC**
**TCGA-BH-A18T**	**0.61(-)**	**0.04(-)**	**29.35**	**2+**	**+**	**-**	**-**	**TNBC**
^∗^ **TCGA-AO-A0JL**	**0.63(-)**	**0.08(-)**	**63.60**	**1+**	**-**	**+**	**+**	**non-TNBC**
TCGA-AN-A0XV	110.33(+)	3.50(+)	22.17	2+	+	-	-	non-TNBC
TCGA-AO-A12C	18.88(+)	3.60(+)	95.67	3+	+	-	-	non-TNBC
^∗^ **TCGA-BH-A18Q**	**2.83(-)**	**1.10(-)**	**20.93**		**+**	**-**	**-**	**TNBC**
TCGA-LL-A5YL	9.84(+)	0.52(-)	55.32	3+	+	-	-	non-TNBC
TCGA-E2-A10A	59.40(+)	9.89(+)	32.99	2+	+	-	-	non-TNBC
TCGA-AO-A03L	14.71(+)	1.35(+)	58.72	2+	+	-	-	non-TNBC
TCGA-AO-A12G	40.39(+)	1.80(+)	45.03	2+	+	-	-	non-TNBC
TCGA-BH-A1EX	5.50(+)	0.28(+)	41.46		+	-	-	non-TNBC
TCGA-BH-A0AU	37.14(+)	16.19(+)	51.79		+	-	-	non-TNBC
^∗^ **TCGA-A2-A04U**	**0.02(-)**	**0.02(-)**	**9.64**	**1+**	**-**	**+**	**+**	**non-TNBC**
TCGA-AN-A0XW	13.96(+)	5.32(+)	22.04	2+	+	-	-	non-TNBC

Individuals marked with asterisks show no concordance regarding HER2 labeling by different testing and are misclassified by logistic regression based on the 3 variables clinically used to classify breast cancer patients into TNBC

Given the larger number of individuals with available HER2 (IHC) *status* (*n*=913) compared to the available HER2 (IHC) level (*n*=619), the HER2 classification provided by IHC *status* was considered. As mentioned before, a second HER2 classification of individuals can be obtained by the FISH method. Given that FISH provides as a more accurate test for classifying individuals into HER2 ‘positive’ or ‘negative’, the HER2 classification of the 417 individuals measured by FISH was taken to replace the classification based on the IHC *status* of the same individuals (IHC + FISH; Tables 2 and 3). This constitutes the baseline classification of the patients that will be further used throughout this study.

Having built the final TNBC dataset, a summary of the expression of ER, PR and HER2 (based on IHC or FISH, whenever available) can be found in Table 4, where it is clear the down-regulation of these TNBC-associated genes in TNBC individuals (class ‘1’). FPKM normalized gene expression data were log-transformed prior to data analysis.

**Table 4:** Summary of FPKM values obtained for ER, PR and HER2 for the individuals under study

	Class	Min.	1st Qu.	Median	Mean	3rd Qu.	Max
ER	0	0.016	16.144	36.667	47.881	69.649	272.203
	1	0.019	0.160	0.351	1.530	0.828	29.979
PR	0	0.008	0.600	4.228	12.012	15.326	327.913
	1	0.001	0.040	0.079	0.712	0.186	22.978
HER2	0	0.605	26.580	38.732	99.741	58.801	1668.353
	1	1.561	13.964	19.776	21.991	26.058	103.68

### Model selection

With the goal of assessing the significance of the gene expression variables used to classify patients into TNBC and non-TNBC, i.e. ER, PR and HER2, a first logistic regression model based on the 3 variables was built. From the TNBC dataset created, three quarters of randomly selected individuals were assigned to training samples (*n*_*train*_=764; 121 TNBC and 643 non-TNBC), whereas the remaining individuals were assigned to test samples (*n*_*test*_=255; 39 TNBC and 216 non-TNBC). The significance of the three TNBC-associated variables in the outcome variable (TNBC vs. non-TNBC), along with the number of misclassifications, were evaluated.

Univariate logistic models accounting for possible confounding effects on the gene expression data were also evaluated. The variables tested for significance were: gender, race, menopause *status*, age at initial pathologic diagnosis, history of neoadjuvant treatment, person neoplasm cancer *status* and event pathologic stage. The significance of the categorical variables was also determined by the Fisher’s exact test. The variables found to be significant on the outcome (TNBC vs. non-TNBC) were taken for further analysis.

Three model selection strategies were chosen for the application of the RP test for outlier detection based on TNBC gene expression data plus the significant clinical variables identified above: i) variable selection by sparse logistic regression using EN regularization, herein called LOGIT-EN; ii) variable selection and feature extraction by SPLS-DA; and iii) variable selection and feature extraction by SGPLS. The optimization of the model parameters based on the mean squared error (MSE) was performed by 10-fold cross-validation (CV) on the full dataset. For LOGIT-EN, varying *α* values (1>*α*>0) were tested; for SPLS-DA and SGPLS, both varying values for *α* and *L* (*l*=1,…,5) were evaluated in the CV procedure. The optimized parameters were used in the final three models. The Cook’s distance was calculated for each observation *i* in each model *j*. The RP test was then applied, as described in the next section.

As the estimated models and, consequently, the outliers detected, are data-dependent, a sampling strategy was designed to determine whether resampling the data using a subset of observations or features (i.e, *feature bagging*) would identify the same outlier observations when compared to using the original data. A TNBC dataset composed of 80*%* observations randomly selected without replacement was thus created. Model classification was performed by logistic regression with EN regularization (*α*=0.7), as shown to provide the lowest MSE among the three models evaluated (later in the “Results and discussion” section). The model predictions and the Cook’s distance for all observations were then obtained. These procedure was repeated 100 times, resulting in 100 models to be accounted for in the RP test.

Following the recent finding that most random gene expression signatures are significantly associated with breast cancer outcome [30], another resampling strategy was adopted. A TNBC dataset composed of all individuals and 1000 randomly selected variables (without replacement) was fit to a logistic regression with EN regularization (*α*=0.7). The procedure was repeated 100 times, resulting in 100 models to feed the RP test.

In both resampling procedures, the goal was to identify the observations consistently classified as influential, independently of the subset of randomly selected samples used for model building or the subset of randomly selected genes. This approach confers robustness to the overall procedure and constitutes an ensemble strategy to deal with the variability and estimation problems.

The models were built using the following R packages: ‘glmnet’ for regularized logistic regression; and ‘spls’ for SPLS-DA and SGPLS.

## Results and discussion

### TNBC data

#### Exploratory analysis

A first logistic regression model based on the 3 variables clinically used to classify patients yielded significance only for variables ER and HER2. A total of 45 and 12 misclassifications were obtained for the train and test sets, respectively, from which 9 are suspect regarding their HER2 label identified above (Tables 2 and 3; marked with asterisks), with 6 corresponding to individuals ER- and PR-, and discordant HER2 label.

When looking for potential confounding variables before getting into outlier detection based on gene expression data, univariate logistic regression and the Fisher’s exact test identified *race* and *age* as significant (*p*<0.05) for the outcome (TNBC vs. non-TNBC). These variables were combined to the gene expression dataset for ensemble outlier detection, as described next.

#### Ensemble outlier detection

Three modeling strategies for dimensionality reduction in the original TNBC dataset were evaluated for independently estimating the individuals’ outlierness based on the Cook’s distance. From the 19690 initial variables, LOGIT-EN, SPLS-DA and SGPLS selected 107, 2945 and 551 variables, respectively, with 26 variables in common (Table 5).

**Table 5:** Ensemble outlier detection results for the TNBC dataset (mean values for the number of variables selected, MSE and misclassifications for the random strategies are presented)

	TNBC original data			Random patients	Random variables
	LOGIT-EN	SPLS-DA	SGPLS	LOGIT-EN	
Variables selected	107	2945	551	82	65
MSE	0.020	0.025	0.084	0.032	0.035
Misclassifications	16	29	23	35	41
Parameter (*α*)	0.9	0.8	0.7	0.7	0.7
Parameter (*L*)	-	4	4	-	-
Resampling number	-			100	100
Influential observations	24			40	37
Suspect (influential) observations	2			6	6

SPLS-DA and SGPLS models accounted for 4 LVs extracted, based on *α* values of 0.8 and 0.7, respectively. LOGIT-EN, with optimum *α*=0.9, yielded better accuracy regarding the MSE obtained, compared to the PLS-based models (Table 5). LOGIT-EN also produced a lower number of misclassifications, i.e., 16, compared to SPLS-DA and SGPLS (29 and 23, respectively).

**Fig. 1:**
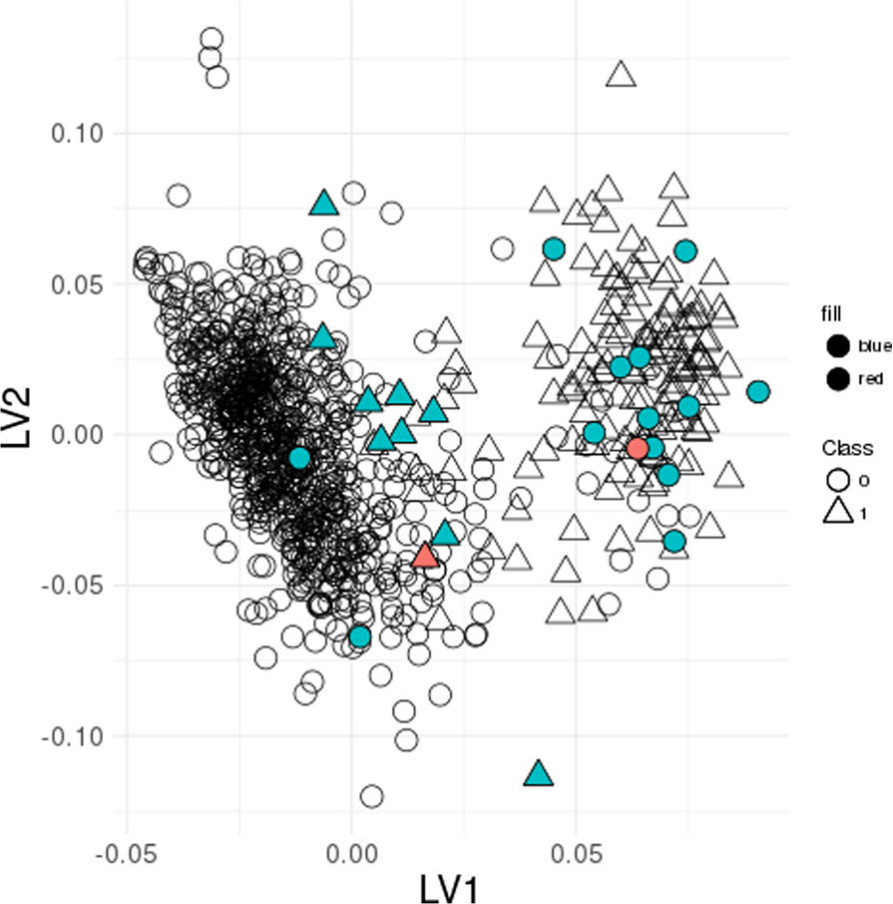
Individuals’ distributions in the space spanned by the first two SPLS-DA latent vectors. Circles, non-TNBC individuals; triangles, TNBC individuals; blue data points are influential observations; red data points are influential observations which are suspect regarding their HER2 label

PLS modeling allows graphically displaying observations in the space of the LVs explaining the largest variance in the data. Such representation in the space of the LVs extracted by SPLS-DA (providing the smallest MSE among PLS-based approaches) can be found in Fig. 1. An overall good separation of TNBC from non-TNBC individuals can be observed.

The individuals’ outlierness ranks by the three modeling strategies were then combined for ensemble outlier detection by the RP test. A total of 24 observations (Table 5) were identified as influential (10 TNBC and 14 non-TNBC), from which 2 correspond to suspect individuals regarding their label (‘TCGA-A2-A0EQ’, TNBC; and ‘TCGA-LL-A5YP’, non-TNBC), as described above (Table 6; Fig. 1). These 2 suspect individuals were previously misclassified by the logistic model based on the three TNBC-associated variables (ER, PR and HER2). By the inspection of Fig. 1 obtained by SPLS-DA, it is interesting to note that all influential observations identified by our ensemble method are placed in the opposite class data cloud, with the exception of 2 non-TNBC (TCGA-E2-A1LB and TCGA-A2-A3XV), corresponding to two non-TNBC samples (blue marks) in the middle of the non-TNBC data cloud, being classified as the actual class by SPLS-DA. Although apparently well classified regarding the measures for three TNBC-associated genes, these individuals might have some abnormal behaviour the the covariate space that make them deviating from the model and, therefore, being highly ranked for outlierness.

**Table 6:** Summary of the 24 individuals identified as influential by the RP test applied to the original TNBC data (individuals highlighted in bold are suspect individuals; asterisks refer to individuals identified as influential for both the original TNBC data and after resampling samples or variables)

Individual	ER	PR	HER2	HER2 level	HER2 *status*	HER2	Type	LOGIT-EN	SPLS-DA	SGPLS	RP	*p*-value	q-value	Miscl.
				(IHC)	(IHC)	(FISH)								(%)
^∗^TCGA-AC-A2QJ	0.10(-)	0.00(-)	7.35		-		TNBC	13	1	34	442	1.40e-05	0.006	67
^∗^TCGA-E9-A22G	0.44(-)	0.02(-)	15.32		+		non-TNBC	6	13	7	546	1.83-05	0.006	33
^∗^TCGA-AR-A251	1.57(+)	0.10(-)	14.02	2+	Equiv	-	non-TNBC	11	5	9	495	1.61e-05	0.006	67
^∗^TCGA-AR-A1AJ	1.47(+)	0.07(-)	9.74		-		non-TNBC	2	10	44	880	3.35e-05	0.007	100
^∗^TCGA-A2-A3Y0	2.18(+)	0.03(-)	11.34	1+	-		non-TNBC	8	7	22	1232	5.01e-05	0.009	33
TCGA-A2-A3XV	0.02(+)	0.03(-)	137.94	2+	Equiv	+	non-TNBC	166	2	5	1660	7.09e-05	0.011	33
^∗^TCGA-E9-A1ND	1.44(-)	0.05(-)	13.05		+		non-TNBC	5	11	48	2640	1.20e-04	0.016	100
TCGA-EW-A1P1	3.02(-)	2.56(-)	23.64	2+	Equiv	-	TNBC	16	24	8	3072	1.43e-04	0.016	100
^∗^TCGA-E2-A1II	0.14(-)	0.19(+)	10.73	1+	-		non-TNBC	9	21	20	3780	1.80e-04	0.017	33
^∗^TCGA-C8-A3M7	4.27(-)	0.76(-)	25.47		-		TNBC	1	28	153	4284	2.06e-04	0.017	100
^∗^TCGA-D8-A1JF	1.26(-)	0.11(-)	32.93	1+	-		TNBC	19	109	2	4142	1.99e-04	0.017	0
TCGA-BH-A42U	9.19(-)	1.83(-)	38.37		-		TNBC	7	3	223	4683	2.28e-04	0.018	100
^∗^TCGA-LL-A740	0.30(-)	0.12(-)	68.56	2+	Equiv	-	TNBC	47	125	1	5875	2.91e-04	0.021	0
^∗^TCGA-A2-A1G6	23.90(-)	21.45(-)	29.74	1+	-		TNBC	10	4	204	8160	4.14e-04	0.025	100
^∗^TCGA-OL-A5S0	0.09(+)	0.06(-)	31.92			+	non-TNBC	46	8	21	7728	3.91e-04	0.026	67
^∗^ **TCGA-A2-A0EQ**	**2.13(-)**	**0.04(-)**	**30.15**	**3+**	**+**	**-**	**TNBC**	**4**	**12**	**216**	**10368**	**5.33e-04**	**0.031**	**67**
^∗^TCGA-A2-A0YJ	0.09(+)	0.03(-)	240.24	0	-		non-TNBC	27	34	12	11016	5.68e-04	0.031	0
^∗^TCGA-AR-A1AH	0.03(+)	0.03(-)	34.12		-		non-TNBC	21	16	38	12768	6.63e-04	0.034	100
^∗^TCGA-AC-A62X	0.19(+)	0.02(-)	28.53				non-TNBC	22	35	19	14630	7.63e-04	0.037	0
TCGA-E2-A1LB	0.42(-)	0.09(-)	1129.87	3+	+		non-TNBC	230	6	11	15180	7.93e-04	0.037	33
^∗^TCGA-OL-A97C	16.25(-)	8.56(-)	24.04			-	TNBC	14	41	30	17220	9.02e-04	0.040	33
^∗^ **TCGA-LL-A5YP**	**0.16(+)**	**0.05(-)**	**15.10**	**1+**	**-**	**+**	**non-TNBC**	**50**	**22**	**18**	**19800**	**1.04e-03**	**0.043**	**100**
TCGA-C8-A26X	0.42(-)	0.13(-)	60.12	1+	-		TNBC	53	64	6	20352	1.07e-03	0.043	33
TCGA-D8-A1XW	0.2(-)	0.11(+)	21.03	1+	-		non-TNBC	23	14	68	21896	1.15e-03	0.044	33

A careful inspection on outlier individuals detected might help disclosing their outlierness nature, as inconsistencies regarding the HER2 (both IHC and FISH) labels of the influential individuals can be observed (Table 6). For instance, individual ‘TCGA-LL-A5Y’, a suspect individual identified as influential, was labeled as HER2+, when its HER2 value most probably indicates negativity for the gene. This individual was classified as HER2- by IHC testing. Moreover, it may happen that its ER+ label is incorrect, given the corresponding ER expression value. Therefore, individual ‘TCGA-LL-A5Y’ might indeed be a TNBC patient. The opposite situation can be observed for patient ‘TCGA-A2-A0EQ’, showing ER and HER2 expression values indicating positivity for the genes (as determined by IHC), as opposed to their negative labels. If properly labeled, this individual would have been initially classified as non-TNBC. Abnormal HER2 expression values regarding their corresponding negative labels were observed for individuals ‘TCGA-A2-A0YJ’ (240.2), ‘TCGA-LL-A740’ (68.6) and ‘TCGA-C8-A26X’ (60.1). This is particularly important for the last two patients, as a correct HER2 label would have result in a classification of non-TNBC instead of TNBC.

Although suspect individuals are only related to the HER2 labels, the ensemble outlier also disclosed potential outliers for ER and PR labels, as seen for the influential, suspect individuals described above. From the influential individuals identified (Table 6), several show ER and PR FPKM values that should correspond to the opposite gene receptor label (‘positive’ or ‘negative’), thus compromising the final TNBC patients’ classification based on the ER, PG and HER2 labels. Besides ‘TCGA-LL-A5Y’ and ‘TCGA-A2-A0EQ’, this is also clear e.g. for individuals ‘TCGA-EW-A1P1’, ‘TCGA-C8-A3M7’, ‘TCGA-BH-A42U’, ‘TCGA-A2-A1G6’ and ‘TCGA-OL-A97C’.

It is noteworthy that our proposed ensemble approach is robust to individual model or specific method inconsistencies. In fact, if only one method is taken into account, some outliers can fail to be identified, whereas by creating and testing a unique ensemble ranking that problem is partially mitigated. For example, patient TCGA-C8-A3M7 is ranked in position 1 using LOGIT-EN, but not identified as an outlier when using SGPLS (rank position 153). There is strong evidence that this patient is an outlier, based on the ER expression value that indicates positivity for the gene, but was labeled as negative in the clinical data. If labeled correctly, this patient would have been originally labeled as non-TNBC (with at least one gene positive). On the other hand, patient TCGA-BH-A42U is identified as an outlier when applying LOGIT-EN and SPLS-DA (rank positions 7 and 3, respectively), but not for SGPLS (rank position 223). Again, the non-concordant ER label with respect to the ER expression might explain the outlierness of this patient. As a final example, patient TCGA-LL-A740 is the most deviating observation for SGPLS (rank position 1), but ranked in positions 47 and 125 for LOGIT-EN and SPLS-DA, respectively. The outlierness of this patient might be due to a wrong HER2 label regarding the HER2 expression. These examples illustrate the fact that each particular method may not be adequate to correctly identify these observations and only an ensemble approach can detect these cases. We hypothesize that adding more methods or criteria may even improve the results, as the bootstrap strategy conducted showed.

The resampling strategies, either applied to the observations (‘Random patients’) or to the variables (‘Random variables’), were able to classify TNBC data with no loss of accuracy regarding the mean MSE (Table 5). The number of misclassifications increased, as expected, as by resampling patients with replacement or with resampling 1000 variables out of the 20,000, some valuable information might be lost. The mean number of variables selected with EN regularization (*α*=0.7) was 82 for the ‘Random patients’ strategy, with 23 selected in more than 75% of the runs, from which 15 were in common with the consensus set of 26 variables selected by the modeling strategies evaluated applied to the original TNBC data (Table 5). For the ‘Random variables’ strategy, the mean number of variables selected was 65, out of the 1000 randomly selected in each run.

A total of 40 and 37 observations were found as influential based on the ‘Random patients’ and ‘Random variables’ strategies, respectively (Table 5). From the influential individuals identified, 6 (the same individuals in both strategies) matched suspect individuals identified above, i.e., individuals with discordant HER2 testing results, including the 2 suspect individuals identified as outliers based on the original TNBC data (Tables 5 and 6). More influential observations were detected upon resampling, which might be due to the fact that the same model, i.e., LOGIT-EN, was being used in the 100 bootstrap runs, therefore achieving more concordant results, as opposed to using three different models in the previous, not resample-based, approach. Consequently, with this increase in the number of influential observations identified, a higher number of suspect individuals identified as outliers is also expected to be obtained.

Overall, from the application of the ensemble outlier detection method, either to the original TNBC dataset or to two TNBC datasets resulting from resampling individuals or gene expression variables, a consensus set of 18 outlier patients was obtained (Table 6; marked with asterisks).

Given the difficulty in validating the outlierness of the influential individuals identified, as no benchmark classification for this dataset was available, six synthetic datasets were created to evaluate the performance of the ensemble method in controlled scenarios. The new datasets were simulated with dimension *N*=1000 (500 observations for each class, ‘1’ and ‘0’) and considering two variable dimensional spaces of normally distributed random variables, of *p*=19688 (full dimensionality of the TNBC dataset) and a subset of randomly selected *p*=5000, both *N*≪*p* scenarios. The mean and covariance matrices from the TNBC and non-TNBC classes were taken for data simulation, in order to simulate a complex, more realistic and less separable scenario. A varying percentage of simulated outliers, i.e., 2.5%, 5% and 10%, was also considered, by randomly changing the label for 25 (2.5%), 50 (5%) and 100(10%) data points, to determine the adequacy of the RP test for identifying true outliers. The three methods previously considered, i.e., LOGIT-EN, SPLS-DA and SGPLS, were used to build the ensemble. The performance of the methods and the ensemble was evaluated based on the number of false positives and false negatives in the top 25, 50 and 100 ranked individuals for outlierness, based on the Cook’s distance and the RP statistic. The selection of the top 25, 50 and 100, respectively, aimed at using the exact (known) values of the outliers. This optimal scenario avoids the use of further selection parameters and prevents the favoring of any of the methods analyzed. In realistic scenarios where the number of outliers is unknown, other strategies could be used, e.g., based on Cook’s distance distribution, with the disadvantage that threshold values would have to be chosen.

For all synthetic scenarios evaluated, the performance of the ensemble method (Additional file 1) was comparable to that of LOGIT-EN (the one identifying more outliers considering the top ranked invididuals), either similar, slightly lower, and in one case better (for *p*=19688 and 10% simulated outliers). It is expected that accounting for less accurate models compromises the RP performance, so it is desired that many models are included in the ensemble, so that less accurate models do not deteriorate the RP results. Even with sub-optimal solutions or as good solutions compared to those obtained by a given method, the use of the ensemble method proposed is highly encourage as a way to evaluating several methods in a fast, automated way, instead of individually optimizing several methods (computational time consuming) and evaluating different cut-off values for the Cook’s distances or any other measure for outlierness chosen. Instead, it provides a statistical assessment of the outlierness of each individual.

**Fig. 2:**
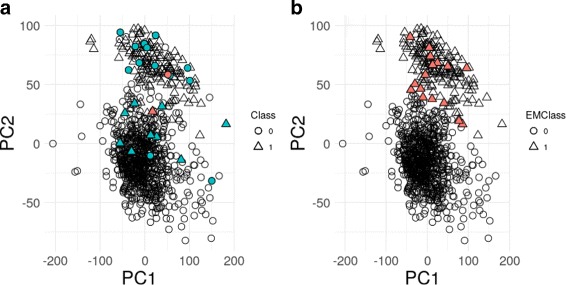
Individuals’ distributions in the space spanned by the first two Principal Components. **a** symbols correspond to actual labels: circles, non-TNBC individuals; triangles, TNBC individuals; blue data points are influential observations; red data points are influential observations which are suspect regarding their HER2 label. **b** symbols correspond to predicted labels by the EM algorithm: circles, non-TNBC individuals; triangles, TNBC individuals; red data points are actual non-TNBC observations, for which at least one of the 3 TNBC-associated genes has an arguably high expression value

In order to compare the proposed ensemble model-based method with non-supervised approaches, we further conducted conducted Principal Component Analysis (PCA). Figure 2a displays the observations in the space of principal components (PC1 and PC2), with influential observations highlighted, and where we can observe that these individuals do not exhibit any abnormal covariate patterns. When superimposing the labels, most outliers previously identified seem to be in the incorrect class, however, the full identification seems to be much more subtle, with some outliers identified placed in the correct class and many other, not identified as outliers, placed in the incorrect class. With no clear-cut measure for outlierness, PCA cannot identify outliers, unless such observations clearly stand out from the data cloud in the covariate space of reduced dimension. The Expectation-Maximization (EM) algorithm was then applied to the first 2 PCs obtained with the goal of obtaining a class membership for each observation. In a graphical representation in the space of the first 2 PCs of the class assigned to each individual (Fig. 2b), even without a clear visual separation of clusters, the clusters obtained are now homogeneous, with no individuals from the wrong predicted class. However, when compared to the actual class labels, a total of 65 observations were clustered in the wrong class. For those individuals, and focusing on the class ‘1’ (TNBC) predicted individuals, for a total of 16 (Fig. 2b; coloured symbols) at least one of the 3 TNBC-associated genes has an arguably high expression value, thus indicating that the corresponding assignment to the TNBC class is wrong. Therefore, our results stand as more meaningful compared to the non-supervised methods evaluated, which reinforces our choice of using supervised model-based classification for outlier detection. Moreover, our ensemble method provides a statistical assessment of individuals’ outlierness, not relying on either subjective visual cut structures, definition of the number of components extracted and clusters created, or predefined cut-off measures of outlierness. In fact, for the PCA strategy one has to choose the number of principal components to use, then decide the clustering algorithm to be applied (with the corresponding associated parameters) and finally thresholds to classify the points.

The proposed ensemble outlier detection approach therefore stands as a very promising tool for outlier detection in high-dimensional ‘omics data, being robust to resampling strategies on both samples and variables. The method can also be easily extended to other modeling strategies and outlierness measures applied to different data structures.

#### Genes selected

From the variables selected by LOGIT-EN, SPLS-DA and SGPLS, 26 genes were selected in common (Table 7). Both race and age were not selected by these dimensionality reduction strategies. Regarding the three TNBC biomarkers, ER was selected as relevant by the three methods, PR only selected by SPLS-DA, and HER2 by SPLS-DA and SGPLS. From the 26 selected variables, 15 (out of 23) were also selected by LOGIT-EN in more than 75% of the runs (Table 7; highlighted in bold), under the ‘Random patients’ ensemble strategy.

**Table 7:** List of up and down-regulated genes in TNBC selected in common by the modeling strategies evaluated on the original TNBC data (variables selected in common with the ‘random patients’ strategy are highlighted in bold)

Up-regulated	***ACE2***	*BPI*	*CLDN10*	*DMRTA2*	***FTDC***	***HAPLN3***	***KCNS1***
	*KIR2DL4*	*KLK6*	***LYPD1***	*MFGE8*	*NRG4*	*PDX1*	***PIF1***
	*POM121L2*	***PPP1R14C***	*SRSF12*	*TAF7L*	***UBASH3B***	*VSNL1*	***ZIC1***
Down-regulated	*ASPN*	***CA12***	***CXXC5***	***ESR1***	***PGAP3***		

In order to evaluate the strength of each variable (gene) within each method, the ranks based on the absolute value of the estimated coefficients were computed, with the lowest rank corresponding to the largest value. The rank levels of the variables selected in common by each method is expected to vary across methods, specially because the number of variables selected is highly different. However, some variables were consistently highly ranked (Additional file 1), e.g., *LYPD1*, and *KCNS1* and *PGAP3*. *LYPD1* was indeed previously reported as up-regulated in TNBC [31].

Next, we interrogated if the genes found as relevant have been already described as differentially expressed in TNBC versus non-TNBC. The majority of genes (21) were up-regulated and 5 down-regulated in TNBC (Table 7). Among the up-regulated genes, only *HAPLN3* [32], *MFGE8* [33], *LYPD1* [31], and *UBASH3B* [34] were already reported as being up-regulated in TNBC, with *LYPD1* the most consensually relevant variable by the three models, as seen above. There were no discrepancies between our data and previously published results. The 5 down-regulated genes in TNBC corresponded to the estrogen receptor (*ESR1*), as expected, and also *CA12*, *ASPN*, *PGAP3* and *CXXC5*. Only *CA12* and *ASPN* were previously reported as being strongly down-regulated in TNBC [35, 36].

As variable selection might be influenced by the presence of outliers, LOGIT-EN, SPLS-DA and SGPLS were applied again to the TNBC dataset, after changing the label of the influential individuals identified by the ensemble method, as described in Section Methods. To evaluate the effect of changing the labels of the most influential observations, we have switched the label of 12 out of the 24 influential observations corresponding to the patients that were misclassified at least by two models out of three models built. Sixteen variables were selected in common by the three models, with eleven (*ESR1*, *TAF7L*, *ACE2*, *CLDN10*, *HAPLN3*, *DMRTA2*, *LYPD1*, *SRSF12*, *PGAP3*, *CXXC5* and *PPP1R14C*) shared with the variables selected when accounting for all patients with the original labels. Some of these variables have been previously reported as TNBC-related, namely, *ESR1*, *HAPLN3* and *LYPD1*, as seen above. An increase in model accuracy in terms of MSE for the three new models was also observed (0.012, 0.010 and 0.078 for LOGIT-EN, SPLS-DA and SGPLS, respectively). From the set of new variables selected, i.e., *NOTO*, *CT83*, *SLCO1B1*, *VSX2* and *TTLL4* (all up-regulated in TNBC), only SNPs of *SLCO1B1* showed significant associations with postmenopausal breast cancer risk, no specific sub-type.[37]

Overall, the biological and pathological role of these genes, previously related to breast cancer, support our method, regarding variable selection. It will be interesting to address the putative role of the remaining genes in breast cancer, in particular TNBC.

## Conclusions

High-dimensional cancer genomic and clinical profiles require appropriate modeling strategies to reduce the complexity of the data while keeping the relevant information to accurately classify patients into their cancer type. In the context of outlier detection, the use of different data reduction and modeling techniques often lead to the identification of different sets of outlier observations, as some of them might be disregarded depending on the model chosen. Here we have shown the potential of ensemble model-based outlier detection for identifying outlier individuals based on their gene expression and clinical variables. The proposed method proved to be robust to resampling strategies, either in patients or variables, providing a consensus set of outlier observations in the framework of high-dimensional omics data and robustly coping with the inherent estimation challenges. The present results represent a valuable tool to significantly improve patient classification regarding their type of cancer and determining which patients should be re-evaluated before any therapeutic decision. Moreover, the model consensus approach leads to the selection of a set of covariates/genes that may be linked to the disease and potentially disclose new therapeutical targets for clinical management of TNBC.

## Additional file

Additional file 1 Ensemble outlier detection and gene selection in triple-negative breast cancer data. (XLSX 4010 kb)
